# Migrant Background and Weight Gain in Early Infancy: Results from the German Study Sample of the IDEFICS Study

**DOI:** 10.1371/journal.pone.0060648

**Published:** 2013-04-04

**Authors:** Anna Reeske, Jacob Spallek, Karin Bammann, Gabriele Eiben, Stefaan De Henauw, Yiannis Kourides, Peter Nagy, Wolfgang Ahrens

**Affiliations:** 1 Leibniz - Institute for Prevention Research and Epidemiology (BIPS), Bremen, Germany; 2 Department of Epidemiology and International Public Health, School of Public Health, Bielefeld University, Bielefeld, Germany; 3 Institute for Public Health and Nursing Research, University of Bremen, Bremen, Germany; 4 Department of Public Health and Community Medicine, University of Gothenburg, Gothenburg, Sweden; 5 Department of Public Health, Ghent University, Gent, Belgium; 6 Research and Education Institute of Child Health, Strovolos, Cyprus; 7 Department of Pediatrics, University of Pécs, Pécs, Hungary; 8 Institute for Statistics, University of Bremen, Bremen, Germany; UCL Institute of Child Health, University College London, United Kingdom

## Abstract

**Objective:**

To examine variations in infant weight gain between children of parents with and without migrant background and to investigate how these differences are explained by pre- and perinatal factors.

**Methods:**

We used data on birth weight and weight at six months from well-child check-up books that were collected from a population-based German sample of children in the IDEFICS study (n = 1,287). We calculated unadjusted and adjusted means for weight z-scores at birth and six months later. We applied linear regression for change in weight z-score and we calculated odds ratios and 95% confidence intervals (95% CI) for rapid weight gain by logistic regression, adjusted for biological, social and behavioural factors.

**Results:**

Weight z-scores for migrants and Germans differed slightly at birth, but were markedly increased for Turkish and Eastern European infants at age six months. Turkish infants showed the highest change in weight z-score during the first 6 months (ß = 0.35; 95% CI 0.14–0.56) and an increased probability of rapid weight gain compared with German infants. Examination of the joint effect of migrant and socioeconomic status (SES) showed the greatest change in weight z-scores in Turkish infants from middle SES families (ß = 0.77; 95% CI 0.40–1.14) and infants of parents from Eastern European countries with high SES (ß = 0.72; 95% CI 0.13–1.32).

**Conclusions:**

Our results support the hypothesis that migrant background is an independent risk factor for infant weight gain and suggest that the onset of health inequalities in overweight starts in early infancy.

## Introduction

There is a large body of evidence on the association of ethnicity, socioeconomic status (SES) and obesity [1]–[4]. In northern European countries, risks for overweight and obesity are elevated especially among immigrants from non-western countries, e.g. Turkey and Morocco, and among people with low SES [5]–[8]. However, research is still needed to understand the mechanisms through which migrant background and socioeconomic status individually and jointly influence the development of obesity over the life course and in which stage of the life course differences in overweight promoting factors emerge. Differences in obesity rates between migrant and non-migrant populations may be caused by genetic factors. Alternatively, migrant background may also be seen as a more general marker for specific social, cultural and behavioural exposures leading to differences in environmental factors. Immigrants experience specific exposures during their life course, which can persist over generations. This may also determine lifestyle and health of the offspring of migrants, starting from intrauterine life and infancy, due to both genetic factors as well as to cultural beliefs and health behaviours transferred from parents to the offspring [9].

The prenatal stage and infancy have been identified as critical periods for the development of overweight and obesity in children [10], [11] with high birth weight and rapid weight gain as risk factors for later obesity, metabolic and cardiovascular risk [12]–[14]. Several reports have shown ethnic and social differences in early growth patterns, e.g. a higher prevalence of rapid growth among infants born to parents with migrant background and low SES compared to other social groups [15]–[18]. Also, the distribution of important determinants of rapid weight gain, such as duration of breastfeeding [19], [20], birth weight [21]–[23] and pre-pregnancy BMI as well as high gestational weight gain [24], [25] varies between ethnic groups, which might contribute to an early start of health inequalities.

The population-based German sample of children in the longitudinal IDEFICS study (“Identification and prevention of dietary- and lifestyle-induced health effects in children and infants”) served as the basis for a historical cohort study to examine differences in infant weight gain during the first 6 months of life between migrant groups and Germans while controlling for biological and maternal behavioral determinants of infant weight gain in early life.

## Methods

### Ethics statement

Ethical approval was obtained from the ethics review board of the University of Bremen. All participating parents or legal guardians gave written informed consent for data collection for themselves and their children. Each child gave oral consent after being orally informed about the modules by a study nurse immediately before examination using a simplified text. All procedures were approved by the ethics review board.

### Study sample and exclusions

Study data were collected in the framework of the IDEFICS study and are governed by the study consortium, which grants access to all study centers who have contributed to their generation. The IDEFICS study is a population-based multicenter intervention and cohort study on prevention and causes of childhood obesity, which includes children aged 2 to 9 years at baseline from 8 European countries. In each of the 8 countries, participants were recruited through kindergarten and school settings in one intervention and one control region and two cross-sectional surveys were conducted [26]. The first, a baseline survey, was conducted between September 2007 and May 2008 and the second, a follow-up survey, from September 2009 to May 2010. Following a standardized protocol, the baseline survey included an extensive examination programme covering standard anthropometric measures, clinical parameters and biomarker (urine, blood and saliva) of children [26]. Parents were also asked to complete a parental questionnaire to assess information on gestational and behavioral characteristics (e.g. smoking during pregnancy, feeding practices), quality of life and familial social circumstances (e.g. income, education and migrant background). The questionnaire was designed as a self-completion questionnaire and was available in several languages, e.g. German, Turkish and Russian. Where required, parents were also offered assistance completing the questionnaire. A more detailed description of the survey has been published elsewhere [26], [27]. In Germany, maternal antenatal care cards and well-child checkup books, which obtain data on weight gain of mother during pregnancy and postnatal weight gain of children, were collected and copied during the examination modules.

**Figure 1:**
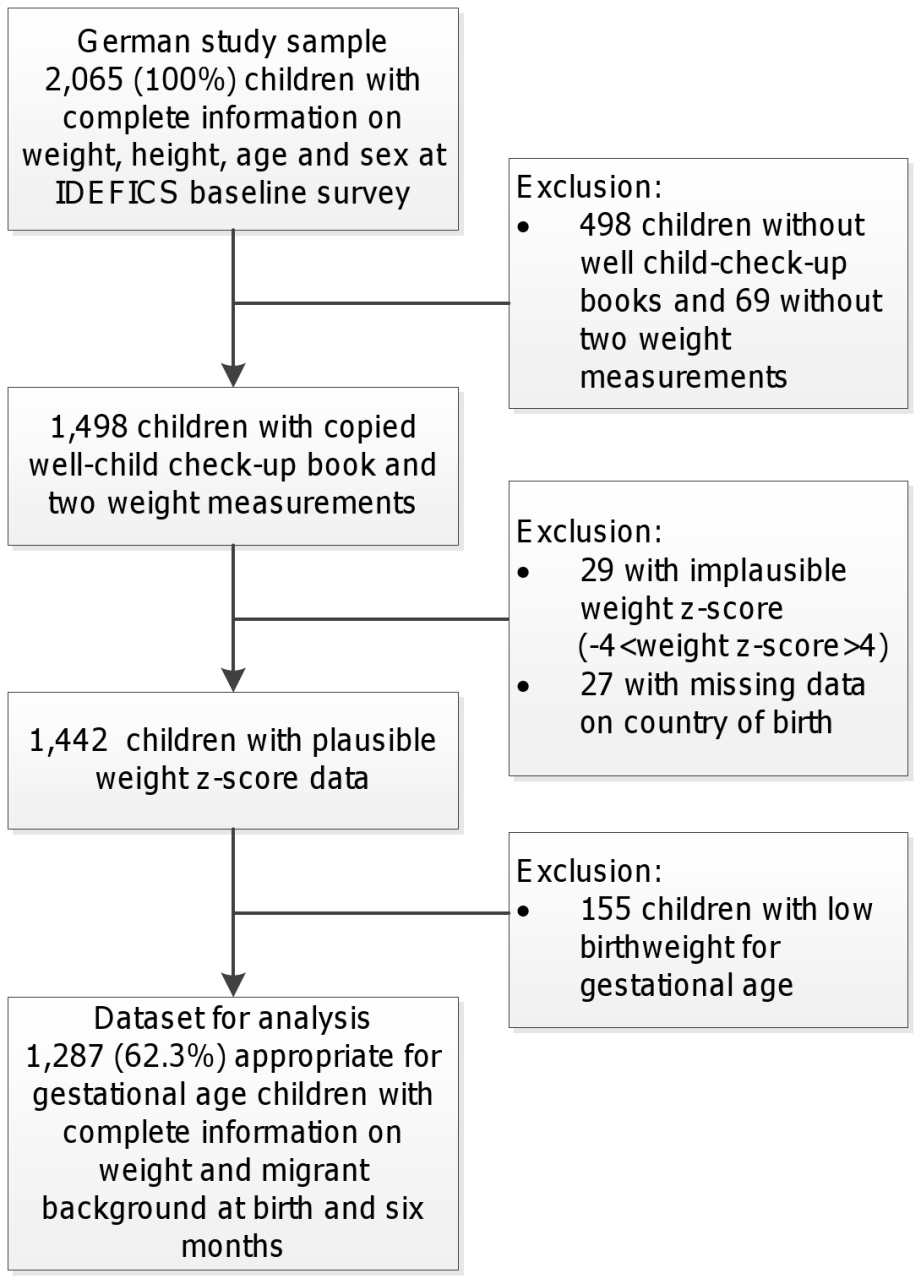
Flow chart describing the exclusion criteria and number of children excluded from analysis.

The present analysis only includes children residing in Germany. The baseline survey in Germany (September 2007 – May 2008) reached a response proportion of 49.8%, with 2,065 eligible cases [26]. Well-child checkup books with weight and height measurements at birth and six months of age were available for 72.7% of the baseline sample. For the present analysis we only included appropriate for gestational age children (AGA) with complete information on migrant background as well as weight at birth and six months (n = 1,287). Reasons for exclusions and the corresponding number of subjects are summarized in Figure 1.

### Measurements

#### Migrant background

Regarding the migration background parents were asked if the child, the mother, father or both parents were born in Germany or elsewhere (with specification of country of birth). A child was defined to have a migrant background if it, or one, respectively both parents were born outside Germany, while a child whose parents were born in Germany was considered as German without a migrant background. We specified three migrant groups: from Turkey, from Eastern European countries (including countries of the former Yugoslavia, former Soviet Union as well as Poland, Romania and Hungary) and other countries which mainly included participants originating from the Lebanon, Kosovo, Iraq and Vietnam. Families with both parents born in foreign countries were categorized according to the maternal country of birth.

#### Socioeconomic status

To assess the socioeconomic status of families, information on parents’ education, income and occupation was collected. We calculated a three-dimensional, additive index to assess socioeconomic status (SES), the ‘Winkler Index’ [28]. This index is commonly used in German social epidemiology studies and allows a categorization into low, medium and high SES categories. We also used parental education for sensitivity analyses. For this purpose, parental education was coded according to the International Standard Classification of Education (ISCED 1997 [29] considering the highest level of education of both parents as well as the highest level of professional qualification.

#### Infant growth

Measurements of weight were derived from well-child checkup books, which record child growth and other health outcomes as regularly monitored by a pediatrician between birth and adolescence. For this analysis we used the measurements of weight at birth and weight at the “U5” examination (6–7 months of age). Data were checked and cleaned. On average infants of German and migrant background were 6.5 months at the U5 examination.

The primary outcomes for our analyses were weight standard deviation scores (z-scores) at birth and six months of life. Age- and sex-specific values were calculated by using the LMS growth macro for Microsoft Excel by Pan and Cole [30] based on the WHO Child Growth Standard for pre-school children from the WHO Multicentre Growth Reference Study [31]. We assessed infant growth as gain in weight z-score between birth and six months of life. We interpreted a positive change as a faster growth than expected, negative change as slower growth than expected. No change of the weight z-score between birth and 6 months was regarded as constant growth within the same centile [17]. Rapid infant weight gain was defined as a > +0.67 change in weight z-score from birth to six months according to [12]. A standard deviation score of 0.67 represents the distance between weight percentiles on standard infant growth charts. Exceeding this limit is interpreted as an upward centile crossing. Weight z-score values <-4 or >4 were considered implausible and excluded from the analysis (n = 29).

#### Covariates

*Exclusive breastfeeding duration* was derived from several questions in the parental questionnaire: ‘What type of feeding was used with your child prior to being fully integrated into the usual household diet?’ including the starting and ending age (in months) of exclusive breastfeeding, combination feeding, formula feeding, and other types of infant feeding (incl. cereals, vegetables, fruit, meat and cow’s milk) and ‘At what age was your child’s diet fully integrated into the usual household’s diet?’. Exclusive breastfeeding was calculated as the period from birth to the earliest stated end of exclusive breastfeeding or the beginning of the introduction of any other food or formula feeding. We categorized exclusive breastfeeding into (1) never breastfed, (2) breastfed exclusively for 1 to 3 months, (3) breastfed exclusively for 4 to 5 months and (4) breastfed exclusively for 6 to 12 months for descriptive analyses, and included exclusive breastfeeding duration as a continuous variable in the regression analyses. *Maternal smoking during pregnancy* was self-reported in the parental questionnaire and classified into three categories: (1) ‘never smoked’, (2) ‘rarely (maximum once a week)’, (3) ‘several times a week or daily’. *Maternal pre-pregnancy BMI and gestational weight gain* were obtained from maternal cards. Maternal pre-pregnancy BMI was classified into overweight/obesity and normal weight/underweight according to the WHO cut-point at 25 kg/m^2^. As maternal pre-pregnancy weight was not recorded in older versions of the maternal card and was therefore not available for the entire study sample, we considered weight at the first antenatal visit as an indicator of pre-pregnancy weight. Self-reported height of the mother was obtained from the parental questionnaire. Gestational weight gain was considered as measured weight at the last prenatal visit minus pre-pregnancy weight. For a measure of adequacy of gestational weight gain, we followed the current guidelines of the Institute of Medicine [32].

*Gestational diabetes* (yes, no) was also obtained from maternal cards and crosschecked with the self-reported information from the parental questionnaire. *Parity* was classified into three categories: (1) ‘0’, (2) ‘1’, (3) ‘2+’. *Age of mother at birth* was included as continuous variable. *Birth weight* of child and *gestational age* were derived from well-child checkup books and included as continuous variables. *Parental height* was self-reported and derived from parental questionnaire.

### Statistical Analysis

Differences in socio-demographic, pregnancy and birth related characteristics between migrant groups and Germans were statistically tested with the chi-square test for categorical variables or student’s t-test for continuous variables. We calculated unadjusted means and 95% confidence intervals (CI) for weight z-score at birth, at six months and for change in weight z-score by migrant background, levels of socioeconomic status, smoking during pregnancy, duration of exclusive breastfeeding, gestational diabetes, maternal pre-pregnancy weight and adequacy of gestational weight gain. Group differences in means of weight z-score where examined by analysis of variance (ANOVA).

We fitted multiple linear regression models to determine the influence of migrant background on change in weight z-score during the first 6 months of life using migrant background as explanatory variable and change in weight z-score as dependent variable, and adjusting for factors known to be associated with weight gain in infancy: parental height, maternal age at birth, pre-pregnancy BMI of the mother, gestational weight gain, gestational diabetes, smoking during pregnancy, birth weight, birth length, gestational age, duration of exclusive breastfeeding and socioeconomic status. First, we tested the full model. Subsequently, we created the final model by backward selection. All covariates with p>0.15 were removed stepwise (model 1). In a second step we conducted the same linear regression analysis replacing socioeconomic status and migrant background by a dummy variable to explore the joint effect of migrant background and SES on change in weight z-score (model 2). To estimate the mean change in weight z-score from birth to six months for the combination of migrant groups and levels of SES, controlling for the covariates mentioned above, we calculated adjusted means from model 2 and used weight z-score at birth as well as weight at six months as dependent variables. We also calculated odds ratios (OR) and associated 95% CI to establish the influence of migrant background on rapid weight gain (z-score >0.67) during the first six months by logistic regression (rapid weight gain yes/no), adjusting for the same factors and using the same model building as described above (model 1 and 2). The model fit was assessed by R^2^, defined as the proportion of variance in weight z-score explained by the independent variables. For the purpose of sensitivity analyses we used parental education (ISCED level) instead of the Winkler-Index. The strength of correlation between ISCED and the Winkler-Index was examined by the Spearman correlation coefficient. All data analyses were conducted using SAS 9.2 (Cary, North Carolina, USA).

## Results

The description of the study population is shown in Table 1. Compared to mothers of children without migrant background, mothers of children with migrant background were of younger age at delivery, more often multiparous and were more often classified as low SES. The gain in weight SD score between 0–6 months was highest among infants of Turkish parents (0.14, 95% CI: 0.02–0.33), while infants from Eastern European parents showed nearly no change in weight z-score (0.04, 95% CI: –0.13; 0.22) and infants from German parents showed a decrease in weight z-score (–0.11, 95% CI: –0.18; –0.04) (Table 2). Overall, 23.8% of all children had a rapid weight gain. After adjustment for biological and maternal behavioral factors (model 1) migrant background was significantly associated with change in weight (p<0.01) with highest effect estimates in infants with Turkish migrant background (Table 3). Also, Turkish infants had a 25% increased probability for rapid weight gain, although this estimate did not reach the level of statistical significance (Table 4).

**Table 1:** Characteristics of study participants included in the analysis (n = 1,287) by country of birth.

	Germany	Turkey	Eastern Europe	Other	p-value*
	(n = 903)	(n = 115)	(n = 145)	(n = 124)	
**Sex of child (% boys)**	51.7	55.7	46.9	49.2	0.52
**Child age at 6 months measurement (months): mean (SD)**	6.5 (0.7)	6.4 (0.9)	6.5 (0.7)	6.4 (0.8)	0.43
**Parental socioeconomic status in %**					
low	20.7	67.7	42.8	46.1	**<0.01**
middle	52.2	28.4	50.0	41.7	
high	27.0	3.9	7.3	12.2	
**Parental education in %**					
ISCED 0–2	28.3	65.4	27.8	45.6	**<0.01**
ISCED 3–4	52.6	25.2	60.4	32.5	
ISCED 5–6	19.2	9.4	11.8	21.9	
**Pre-pregnancy BMI in %**					
Underweight/normal	64.2	59.5	68.3	61.5	0.63
Overweight/obese	35.8	40.5	31.7	38.5	
**Parity (incl. the index child) in %**					
0	47.3	18.0	46.2	38.8	**<0.01**
1	36.9	34.6	34.9	32.7	
2+	15.7	47.4	18.9	28.6	
**Smoking during pregnancy in %**					
never	76.3	70.4	76.2	76.9	0.82
rarely (once a month or less)	5.2	6.5	7.0	5.8	
several times a week or daily	18.5	23.2	16.8	17.4	
**Gestational weight gain (kg): mean (SD)**	14.3 (5.4)	12.5 (5.2)	15.2 (5.9)	14.0 (5.6)	0.01**
**Adequacy of GWG (IOM) in %**					
<ideal weight gain	19.4	36.1	12.1	17.7	**<0.01**
ideal weight gain	36.1	38.9	42.4	30.6	
>ideal weight gain	44.5	25.0	45.5	51.8	
**Gestational diabetes in %**	4.5	5.2	4.8	4.8	0.99
**Age of mother at birth (years): mean (SD)**	29.8 (5.1)	27.5 (5.2)	26.7 (5.4)	28.4 (5.7)	**<0.01**
*Child's birth characteristics*					
**Gestational age (weeks): mean (SD)**	39.2 (1.6)	39.3 (1.7)	39.4 (1.6)	39.0 (1.7)	0.60
**Birth weight (g): mean (SD)**	3508 (466.8)	3451 (427.0)	3524 (476.6)	3404 (443.3)	0.19
**Birth height (cm): mean (SD)**	52.1 (2.4)	51.8 (2.3)	52.3 (2.7)	51.3 (3.0)	0.03*
**Birth weight category in %**					
<3000 g	11.2	11.3	9.7	15.3	0.66
3000–4000 g	76.0	78.3	76.6	75.8	
>4000 g	12.9	10.4	13.8	8.9	
**Duration of exclusive breastfeeding in %**					
Never	10.7	11.9	11.0	9.7	0.15
≤3 months	42.3	47.5	45.6	34.5	
4–5 months	19.2	13.9	25.0	26.6	
≥6 months	27.9	26.7	18.4	29.2	
**Height of mother (cm): mean (SD)**	168.3 (7.6)	162.3 (5.3)	165.0 (7.3)	165.1 (7.1)	**<0.01**
**Height of father (cm): mean (SD)**	182.2 (7.3)	172.9 (5.4)	178.3 (6.6)	175.7 (7.3)	**<0.01**

* p-value from global F-Test (ANOVA) for continuous variables, chi-square test for categorical variables.

** Scheffé test: Gestational weight gain differs between Turkey and Eastern Europe (p<0.05).

Birth height differs between Eastern Europe and Other (p<0.05).

Age of mother at birth different for all migrant groups compared to Germans (p<0.05).

**Table 2:** Weight at birth, weight at six months, change in weight status during first six months (standard deviation and percent of infants with rapid growth by potentially influencing factors.

		Birth	6 months	Change in weight	Rapid growth
	n	z-score (95% CI)	z-score (95% CI)	z-score (95% CI)	%
**Overall**	1287	0.38 (0.33; 0.43)	0.33 (0.27; 0.38)	–0.06 (–0.11; 0.00)	23.8
**Country of birth**					
Turkey	115	0.29 (0.13; 0.45)	0.42 (0.27; 0.58)	0.14 (0.02; 0.33)	21.7
Eastern European countries	145	0.45 (0.29; 0.61)	0.49 (0.35; 0.64)	0.04 (–0.13; 0.22)	22.1
Other	124	0.21 (0.04; 0.37)	0.23 (0.04; 0.42)	0.02 (–0.17; 0.21)	26.6
Germany	903	0.41 (0.35; 0.47)	0.30 (0.24; 0.36)	–0.11 (–0.18; –0.04)	23.9
p-value*		0.07	0.06	0.05	
**Parental socioeconomic status**					
Low SES	366	0.27 (0.18; 0.37)	0.35 (0.24; 0.45)	0.07 (–0.04; 0.18)	27.3
Middle SES	612	0.41 (0.33; 0.48)	0.33 (0.26; 0.40)	–0.08 (–0.17; 0.01)	24.0
High SES	269	0.49 (0.38; 0.59)	0.30 (0.18; 0.42)	–0.18 (–0.30; –0.06)	18.6
p-value*		0.01	0.86	0.01	
**Smoking during pregnancy**					
No	954	0.45 (0.39; 0.51)	0.30 (0.24; 0.36)	–0.15 (–0.22; –0.08)	20.4
Yes	304	0.17 (0.06; 0.28)	0.42 (0.31; 0.54)	0.25 (0.12; 0.39)	35.5
p-value*		<0.01	0.05	<0.01	
**Duration of exclusive breastfeeding**					
Never	130	0.23 (0.09; 0.36)	0.46 (0.28; 0.64)	0.23 (0.03; 0.44)	33.1
≤3 months	514	0.31 (0.22; 0.39)	0.38 (0.30; 0.46)	0.08 (–0.01; 0.17)	27.2
4–5 months	244	0.54 (0.43; 0.64)	0.30 (0.18; 0.41)	–0.24 (–0.36; –0.12)	16.8
≥6 months	326	0.45 (0.34; 0.56)	0.17 (0.06; 0.28)	–0.28 (–0.40; –0.16)	19.6
p-value*		<0.01	0.01	<0.01	
**Maternal pre-pregnancy BMI**					
Normal weight	644	0.33 (0.26; 0.40)	0.31 (0.23; 0.38)	–0.02 (–0.10; 0.06)	24.5
Overweight/ obese	362	0.47 (0.37; 0.57)	0.35 (0.26; 0.45)	–0.11 (–0.23; 0.00)	22.1
p-value*		0.03	0.46	0.19	
**Adequacy of GWG (IOM)**					
<ideal weight gain	185	0.03 (–0.11; 0.17)	0.19 (0.05; 0.32)	0.16 (0.00; 0.31)	28.7
ideal weight gain	342	0.35 (0.26; 0.45)	0.35 (0.25; 0.45)	0.00 (–0.11; 0.11)	24.9
>ideal weight gain	410	0.53 (0.43; 0.62)	0.38 (0.28; 0.47)	–0.15 (–0.25; –0.04)	21.7
p-value*		<0.01	0.08	<0.01	
**Gestational diabetes**					
No	1227	0.38 (0.33; 0.43)	0.32 (0.27; 0.38)	–0.06 (–0.12; 0.00)	23.2
Yes	60	0.38 (0.12; 0.64)	0.40 (0.15; 0.66)	0.02 (–0.25; 0.29)	35.0
p-value*		0.99	0.54	0.58	

* p-value from global F-test in ANOVA.

**Table 3:** Linear regression effect estimates (β) for influence of migrant background on infant weight gain (weight z-score) during first six months of life, adjusted for covariates (n = 1,031).

	Model 1 (R^2^ = 0.34)
	β (95% CI)
**Country of birth**	
Turkey	0.35 (0.14; 0.56)
Eastern European countries	0.28 (0.10; 0.45)
Other	0.23 (0.03; 0.43)
Germany	1.0 (Ref.)
**Gestational age (weeks)**	–0.10 (–0.14; –0.06)
**Birth weight (kg)**	–1.06 (–1.20; –0.91)
**Height of father (cm)**	0.01 (0.00; 0.02)
**Height of mother (cm)**	0.01 (0.01; 0.02)
**Smoking during pregnancy**	
Never	1.0 (Ref.)
Rarely–daily	0.18 (0.04; 0.31)
**Duration of exclusive breastfeeding (months)**	–0.05 (–0.07; –0.03)

**Table 4:** Odds ratios (and 95% CI) for influence of migrant background on rapid weight gain between birth and six months of life, adjusted for covariates (n = 1,031).

	Model 1 (R^2^ = 0.19)
	OR (95% CI)
**Country of birth**	
Turkey	1.25 (0.65; 2.42)
Eastern European countries	1.10 (0.62; 1.95)
Other	1.74 (0.97; 3.09)
Germany	1.0 (Ref.)
**Gestational age (weeks)**	0.80 (0.71; 0.90)
**Birth weight (kg)**	0.14 (0.08; 0.23)
**Height of father (cm)**	1.03 (1.01; 1.06)
**Height of mother (cm)**	1.02 (1.00; 1.04)
**Smoking during pregnancy**	
Never	1.0 (Ref.)
Rarely-daily	1.32 (1.07; 1.62)
**Duration of exclusive breastfeeding (months)**	0.91 (0.85; 0.97)

### Influence of breastfeeding, smoking and socioeconomic status

A longer duration of exclusive breastfeeding was associated with a smaller change in weight and a decreased probability of gaining weight rapidly after birth. Infants of mothers who smoked during pregnancy had a smaller weight z-score at birth than children of non-smoking mothers. The probability of rapid weight gain after adjustment for several covariates was increased by 32% for infants from smoking mothers (Table 4). With regard to household socioeconomic status, infants from low SES families had a lower weight z-score at birth compared to high SES infants and a higher change in weight z-score from birth to six months (0.07, 95% CI –0.04–0.18) (Table 2). However, the observed increased OR for rapid weight gain in the univariate analysis was not statistically significant anymore in the multiple model, after adjustment for smoking and duration of exclusive breastfeeding. Further analyses showed that the majority of high SES mothers exclusively breastfed for 6 months and more compared to only 13% in the low SES group. Also, the low SES mothers showed a considerably higher smoking prevalence (data not shown).

### Migrant background and socioeconomic status

**Figure 2:**
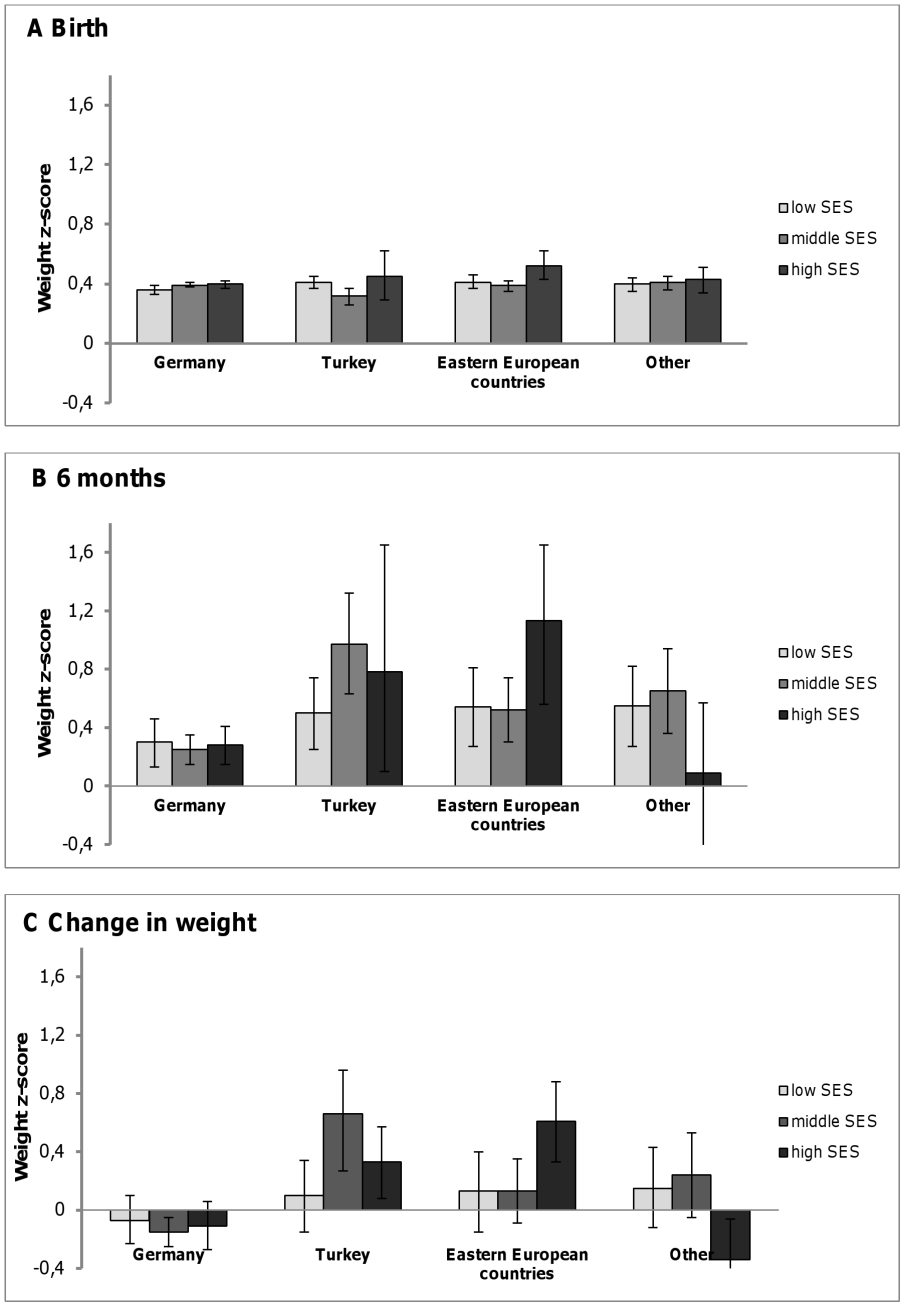
Adjusted mean weight z-scores and 95% CIs according to migrant background and SES. (mean weight z-score adjusted for birth weight, gestational age, parental height, smoking during pregnancy, duration of exclusive breastfeeding).

The joint effect of migrant background and SES on infant growth is shown in Table 5 and the adjusted mean values of weight z-score in migrant and German infants across SES groups in Figure 2a–c. The increase of weight z-score among Turkish infants was particularly obvious in the higher social groups. The highest increase in weight z-score was observed among infants with a Turkish migrant background in the middle SES group and infants with an Eastern European migrant background in the high SES group. Similar trends were found for rapid weight gain (Table 6).

**Table 5:** Linear regression effect estimates (β) for influence of migrant background and parental socioeconomic status on infant weight gain (weight z-score) during first six months of life, adjusted for covariates (n = 1,009).

	Model 2 (R^2^ = 0.35)
	β (95% CI)
Country of birth* parental SES	
Turkey*low SES	0.21 (–0.07; 0.49)
Turkey*middle SES	**0.77 (0.40; 1.14)**
Turkey*high SES	0.44 (–0.57; 1.45)
East. Europe*low SES	0.24 (–0.06; 0.53)
East. Europe*middle SES	0.24 (–0.01; 0.50)
East. Europe*high SES	**0.72 (0.13; 1.32)**
Other*low SES	0.26 (–0.04; 0.56)
Other*middle SES	**0.36 (0.05; 0.67)**
Other*high SES	–0.23 (–0.74; 0.29)
Germany*low SES	0.05 (–0.16; 0.25)
Germany*middle SES	–0.04 (–0.18; 0.11)
Germany*high SES	1.00 (Ref.)
**Gestational age (weeks)**	–**0.10 (**–**0.14;** –**0.06)**
**Birth weight (kg)**	–**1.05 (**–**1.19;** –**0.90)**
**Height of father (cm)**	**0.01 (0.00; 0.02)**
**Height of mother (cm)**	**0.01 (0.01; 0.02)**
**Smoking during pregnancy**	
Never	1.0 (Ref.)
Rarely-daily	**0.15 (0.05; 0.29)**
**Duration of exclusive breastfeeding (months)**	–**0.05 (**–**0.07;** –**0.03)**

**Table 6:** Odds ratios (and 95% CI) for influence of migrant background and parental socioeconomic status on rapid weight gain between birth and six months of life, adjusted for covariates (n = 1,009).

	Model 2 (R^2^ = 0.19)
	OR (95% CI)
Country of birth* parental socioeconomic status	
Turkey*low SES	0.98 (0.44; 2.17)
Turkey*middle/high SES	1.86 (0.66; 5.21)
East. Europe*low SES	0.89 (0.36; 2.21)
East. Europe*middle/high SES	1.19 0.59; 2.38)
Other*low SES	1.52 (0.67; 3.46)
Other*middle/ high SES	1.87 (0.89; 3.94)
Germany*low SES	0.86 (0.50; 1.49)
Germany*middle/ high SES	1.00 (Ref.)
**Gestational age (weeks)**	0.79 (0.70; 0.89)
**Birth weight (kg)**	0.14 (0.08; 0.23)
**Height of father (cm)**	1.03 (1.01; 1.06)
**Height of mother (cm)**	1.02 (0.99; 1.04)
**Smoking during pregnancy**	
Never	1.0 (Ref.)
Rarely-daily	1.32 (1.07; 1.64)
**Duration of exclusive breastfeeding (months)**	0.90 (0.85; 0.96)

### Sensitivity analyses with ISCED

Sensitivity analyses using parental education (ISCED levels) as indicator of SES showed similar results for German and Turkish infants, although in Turkish infants the increase in adjusted weight z-score from birth to six months was lower when adjusting for ISCED instead of the Winkler-Index. The Spearman correlation coefficient between ISCED and the Winkler-Index was 0.56. The correlation was strong among German infants (0.61), moderate among Turkish infants (0.49) and weak among infants from Eastern Europe (0.21). We observed a higher increase in adjusted weight z-scores among low SES infants from Eastern Europe when using the ISCED classification, and no change instead of an increase among infants in the high status group (data not shown).

## Discussion

In our study sample mean weight standard deviation scores for migrants and Germans differed slightly at birth, but were markedly increased at six months for infants from parents originating from Turkey or Eastern European countries. Children with Turkish migrant background showed the highest increase in weight z-score from birth to six months. After adjustment for parental biological and maternal behavioral factors, there was still a significant effect of migrant background on change in weight and rapid weight gain. Examining the combination of migrant background and SES resulted in a particular increase in weight z-score in the higher social groups among Turkish and Eastern European infants, respectively.

Strengths of the present study are the historical prospective perspective on weight development among different social groups, the inclusion of several prenatal and postnatal risk factors for rapid weight gain, as well as the examination of the joint effect of migrant background and SES on infant weight development. We were able to use historical measurements of infant weight, maternal weight and gestational weight gain instead of self-reported data. Since infant weight and maternal weight during pregnancy were assessed prior to our study and independently from the outcome, these data are not affected by recall bias. Although the German IDEFICS sample includes a large proportion of families with migrant background and low SES, numbers of migrants of high SES were small, and results therefore have to be interpreted with caution. Limitations include the response proportion of only 49.8%, which may be of concern with respect to the possibility of selection bias. It is known from other studies that participation is lower in people with low SES and in migrant groups. As we did not solicit systematic information on non-responders, we compared our study population with the population-representative study population of the German KiGGS study with respect to socio-demographic variables [33]. Our sample included a rather large percentage of parents with migrant background (30% compared to 25% in the KiGGS study), while the percentage of families with low SES was similar (29% vs. 28%). However, the distribution of basic characteristics in the migrant and non-migrant group regarding SES and sex of child were comparable with the numbers in the KiGGS study [34]. As our main results were stratified by migrant background and SES, this minimizes the possibility of bias in these two subgroups.

Migrant families where defined by child’s and parental country of birth. However, as we did not collect information on country of birth of grandparents, we might have underestimated migrant background. Parents born in Germany might have been second generation migrants. Future studies should therefore also assess the possibility of second generation migrants, e.g. by asking for country of birth of grandparents.

We used a multidimensional index as indicator of SES in order to avoid residual confounding by unobserved socioeconomic circumstances [35]. When using the one-dimensional ISCED indicator, we observed a higher increase in adjusted weight z-score in the low SES group among infants from Eastern European families and no change instead of an increase in the high SES group. This was due to the fact that in the Eastern European group more than 55% of low SES infants according to the multidimensional index were categorized into the middle or high ISCED level. As these two conventional indicators for SES are apparently not comparable in certain migrant groups and change the interpretation of ethnic differences in infant weight gain, we may need to consider adapted SES indicators for migrant research, taking account of the historical antecedents, the current social circumstances, as well as the different meaning of single indicators of socioeconomic position for different migrant groups [36], [37].

Gain in weight z-scores was calculated using the latest WHO reference for pre-school children, regardless of ethnic or socioeconomic background [31]. Although a German population is not included in this reference, this should not affect the results as we used the z-score only for relative comparisons.

Our results are supported by similar findings from the literature. The Dutch multi-ethnic ABCD study suggested a faster weight gain in the first six months among Turkish and Moroccan infants compared to Dutch children [15] and an increase of these differences during the first years of life [16], which largely contributed to a higher odds of being overweight at age 2 years [38]. Other US and European studies also reported ethnic differences in early weight gain [20], [25], [39]. To the authors’ knowledge, there is no current study looking at the joint effect of socioeconomic status and migrant background on early infancy weight gain so far. A recent publication from the international IDEFICS survey observed higher overweight risks among migrants as well as a socioeconomic gradient in overweight in the majority of European study countries [40]. In our analysis the highest increase in infant weight was observed among migrant children from families with high SES. However, among low SES families, migrant children also had higher weight z-score at six months and a greater change from birth compared with German children. Herngreen and co-authors [18] showed that children of Mediterranean parents in the low-SES group gained significantly more weight and also had higher weight at age 1 and 2 years compared to children of Dutch parents in the low-SES group. The authors assumed that differences might partly be due to differing feeding practices, e.g. an earlier start of combined feeding among migrants.

A longer duration of (exclusive) breastfeeding has been reported to have a protective effect on rapid weight gain and risk of overweight in childhood and later life [41]–[43]. Evidence on socioeconomic and culture-specific differences in infant feeding methods exists, suggesting that more “traditional” mothers within ethnic minority groups as well as more advantaged mothers are more likely to breastfeed their children [19], [20], [44], [45]. The latter likely explains the observed reduction in the association between SES and infant weight gain when adjusting for exclusive breastfeeding. This effect was also shown in a recent study from the UK [26]. In contrast to other studies reporting higher breastfeeding rates or longer duration of exclusive breastfeeding among migrant women [20], [43], our results showed lower initiation rates and duration of exclusive breastfeeding among Turkish mothers. But similar to results from a nationwide Dutch study [20], we found that Turkish mothers with high SES breastfed less than Turkish mothers with low SES, whereas duration of exclusive breastfeeding among German mothers was longest among women with higher status levels. In Turkey as well as other non-industrialized countries it was also observed that highly educated women breastfeed less than women with low education [46]. Thus migrant women of Turkish origin seem to reflect the behavior of women living in their home country. Furthermore, socioeconomic or cultural differences might occur in the type and quantity of food that is introduced after exclusive breastfeeding which might contribute to a faster infant weight gain. An early termination of exclusive breastfeeding among Turkish women is often followed by early introduction of solid food, which might be explained by the culturally-related attitude that a ‘chubby’ child is a healthy child and represents wealth and good motherhood [38], [39]. These early nutritional factors likely have an impact on weight development and the risk of early child overweight.

Modifiable intrauterine risk factors were identified to influence postnatal weight gain in infants, e.g. gestational diabetes, maternal pre-pregnancy BMI and maternal gestational weight gain [47]–[49]. Studies suggest that children born to diabetic or obese mothers or to those with excessive gestational weight gain are more likely to have a higher birth weight for gestational age and a higher risk of overweight in later life. In this study we found the lowest gestational weight gain, but the highest proportion of women with a high pre-pregnancy BMI among Turkish women. In line with these other studies, gestational diabetes, maternal overweight or obesity as well as excessive weight gain during pregnancy resulted in higher infant birth z-scores, but neither showed a clear association with change in infant weight. However, other studies also demonstrated that maternal weight might contribute to ethnic differences in overweight in childhood rather than infancy [24], [32].

## Conclusion

In conclusion, our results support the hypothesis that migrant background is an independent risk factor for infant weight gain and suggest that the onset of health inequalities in overweight lies in early infancy. We found an increased weight z-score from birth to six months of life among infants of parents originating from Turkey and Eastern European countries, especially in the higher SES groups. Termination of exclusive breastfeeding during the first 4 months and early introduction of solid food among Turkish children of high SES might be important factors for high weight gain. In line with other studies, several risk factors in utero, such as gestational diabetes and excessive weight gain of the mother had an influence on rapid weight gain of the child. However, they did not explain the ethnic differences in rapid weight gain observed in this study.
